# Depletion of EREG enhances the osteo/dentinogenic differentiation ability of dental pulp stem cells via the p38 MAPK and Erk pathways in an inflammatory microenvironment

**DOI:** 10.1186/s12903-021-01675-0

**Published:** 2021-06-21

**Authors:** Ran Ran, Haoqing Yang, Yangyang Cao, Wanhao Yan, Luyuan Jin, Ying Zheng

**Affiliations:** 1 Laboratory of Molecular Signaling and Stem Cells Therapy, Beijing Key Laboratory of Tooth Regeneration and Function Reconstruction, Capital Medical University School of Stomatology, Beijing, China; 2 Department of Endodontics, Capital Medical University School of Stomatology, Beijing, China; 3 Department of General Dentistry and Integrated Emergency Dental Care, Capital Medical University School of Stomatology, Beijing, China

**Keywords:** Epiregulin (EREG), Dental pulp stem cells (DPSCs), Osteo, Dentinogenic differentiation, TNF-α, Inflammatory environment

## Abstract

**Background:**

Epiregulin (EREG) is an important component of EGF and was demonstrated to promote the osteo/dentinogenic differentiation of stem cells from dental apical papilla (SCAPs). Whether EREG can stimulate the osteo/dentinogenic differentiation of dental pulp stem cells (DPSCs) in inflammatory environment is not clear. The purpose of the present study is to investigate the role of EREG on the osteo/dentinogenic differentiation ability of DPSCs in inflammatory environment.

**Methods:**

DPSCs were isolated from human third molars. Short hairpin RNAs (shRNAs) were used to knock down EREG expression in DPSCs. Recombinant human EREG (rhEREG) protein was used in the rescue experiment. TNF-α was employed to mimic the inflammatory environment in vitro. Alkaline phosphatase (ALP) staining, Alizarin red staining, quantitative calcium analysis, and real-time RT-PCR were performed to detect osteo/dentinogenic differentiation markers and related signalling pathways under normal and inflammatory conditions.

**Results:**

EREG depletion promoted the ALP activity and mineralization ability of DPSCs. The expression of BSP, DMP-1, and DSPP was also enhanced. Moreover, 50 ng/mL rhEREG treatment decreased the osteo/dentinogenic differentiation potential of DPSCs, while treatment with 10 ng/mL TNF-α for 4 h increased the expression of EREG in DPSCs. Conversely, EREG knockdown rescued the impaired osteo/dentinogenic differentiation ability caused by TNF-α treatment. Further mechanistic studies showed that EREG depletion activated the p38 MAPK and Erk signalling pathways in DPSCs under normal and inflammatory conditions.

**Conclusions:**

Our results demonstrated that EREG could inhibit the osteo/dentinogenic differentiation potential of DPSCs via the p38 MAPK and Erk signalling pathways. Under inflammatory environment, EREG depletion enhanced osteo/dentinogenic differentiation potential of DPSCs by improving the expression of p-p38 MAPK and p-Erk.

**Supplementary Information:**

The online version contains supplementary material available at 10.1186/s12903-021-01675-0.

## Background

Pulpitis is a common disease that is associated with nonvital and missing teeth. Traditional treatment methods include vital pulpotomy, apexification, revascularization, and root canal treatment; however, none of these approaches can solve problems such as cessation of root development, fragile root canal walls and associated complications [1]. Therefore, it is of great significance to find ways to regenerate the dentin-pulp complex in order to maintain normal physiological function. With the development of tissue engineering, increasing research has shed light on the use of mesenchymal stem cells (MSCs) for dentin-pulp regeneration [2]. Based on tissue engineering methods, MSCs can be engrafted to the sites of injury and inflammation; however, the engraftment efficiency is largely influenced by the local microenvironment, with inflammation being one of the most important factors affecting the regeneration efficiency of MSCs for pulpitis [3].

Both trauma and infection, which result in pulpitis, can lead to an inflammatory microenvironment characterized by the accumulation of inflammatory cells, which release proinflammatory cytokines, including tumour necrosis factor-α (TNF-α) [4, 5]. In the acute inflammatory phase, TNF-α has been documented to stimulate the immunosuppressive ability of MSCs [6]. TNF-α was also shown to affect osteoclastogenesis and bone formation [7]. Studies have demonstrated increased apoptotic signalling with compromised longevity of dental pulp stem cells (DPSCs) upon short-term exposure to inflammatory factors [8]. In addition, weak and hyperproliferative DPSCs with diminished mineralization potential were observed in the diseased pulpal tissue [9]. Consequently, it is critical to study the functional changes in DPSCs and to identify suitable growth factors to promote the function of DPSCs in the inflammatory microenvironment.

Recently, researchers have attempted to identify the underlying growth factors and signalling pathways involved in the inflammatory microenvironment. Epidermal growth factor (EGF) has been reported to be a potent regulator [10]. The EGF family comprises multiple mediators, such as transforming growth factor-a (TGF-α), amphiregulin, heparin binding-EGF, and epiregulin (EREG), which are critically involved in regulating fundamental functions in mammalian cells, including survival, migration, and proliferation [11]. In addition, previous studies indicated the important role of EGF family members, such as amphiregulin [12], HB-EGF [13], TGF-α [14], and especially EREG, in inflammation [15, 16]. Similar to the other members of the EGF family, EREG binds to several EGF receptors and thus couples to numerous signalling cascades, most notably the MAPK kinase (Erk) signalling pathway, the phospholipase C gamma pathway, and the PI3 kinase/Akt signalling pathway, to regulate a series of cellular physiological and pathological functions [17], such as vascular remodelling [18], liver regeneration [19] and cutaneous wound healing [20]. Our previous studies showed that EREG could improve the proliferation and osteo/dentinogenic differentiation ability of stem cells from dental apical papilla (SCAPs) by promoting the phosphorylation of MAPK-ERK kinase (MEK)/Erk and c-Jun N-terminal-kinase (JNK) [21, 22]. In addition, EREG improved the migration and chemotaxis ability of adipose-derived stem cells (ADSCs) through the activation of MAPK signalling pathways, including p38 MAPK, JNK, and Erk1/2 [23]. Other studies have also demonstrated that EREG participated in the development of bronchial infection and rheumatoid arthritis [24]. Targeting EREG may provide an effective way to control the progress of infection [25]. In addition, EREG plays a role in skin inflammation and cutaneous wound healing by regulating the Toll-like receptor pathway and angiogenesis [20, 26]. However, it is unclear whether EREG has an effect on MSC function in an inflammatory microenvironment.

In the present study, we aimed to investigate the role of EREG on MSC function in inflammatory microenvironment. DPSCs are the first dental MSCs isolated from the dental pulp of permanent teeth and contribute to dentinogenesis and show good performance in colony formation, survival time, and osteo/dentinogenic ability [27]. Hence, DPSCs are considered an ideal tool to regain lost dental tissues and to re-engineer the root canal system. Therefore, in this study, DPSCs were used to investigate the function of and mechanism of EREG. We used TNF‐α to mimic the inflammatory environment and performed loss- and gain-of-function assays to investigate the role of EREG on the osteo/dentinogenic differentiation ability of DPSCs. Our results demonstrated that EREG displayed a significant role in regulating the osteo/dentinogenic differentiation potential of DPSCs in normal and inflammatory environment through the MAPK signalling pathway.

## Methods

### Human DPSC culture

Wisdom teeth were obtained from Beijing Stomatological Hospital, Capital Medical University, and all procedures were approved by Ethical Committee Agreement, Beijing Stomatological Hospital Ethics Review No.2011-2012). Informed consent was obtained from all patients. Cells were cultured and verified according to our previous study. (22,26) DPSCs were grown in a humidified, 5% CO2 incubator at 37℃ by using Eagle’s medium (Invitrogen, Carlsbad, CA) supplemented with 15% fetal bovine serum (Invitrogen), 2 mmol/L glutamine, 100U/mL penicillin, and 100 mg/mL streptomycin (Invitrogen). The culture medium was changed every 3 days. In the present study, 10 ng/mL TNF-α (R&D Systems, Minneapolis, MN, USA) and 50 ng/ml recombinant human EREG (rhEREG) protein (Abcam, Cambridge, MA, USA) were used to stimulate DPSCs. The p38 MAPK‐specific inhibitor SB203580 (MedChemExpress, NJ, USA) and the Erk‐specific inhibitor PD98059 (Merck, Darmstadt, Germany) were used to study the mechanism of EREG on DPSCs.

### Plasmid construction and viral infection

Plasmid construction and viral infection procedures were carried out as described in our previous study [22]. For viral infections, DPSCs were plated overnight and then infected with retroviruses in the presence of 6 mg/mL polybrene (Sigma–Aldrich, St. Louis, MO, USA) for 6 h. After 48 h, infected cells were selected with 2 mg/mL puromycin for 3 days anddetected the efficacy of shRNA against EREG.Scramble shRNA (Addgene, Cambridge, MA, USA) was used as a control. The target sequences for the shRNAs were as follows:

EREG shRNA: 5′-actactgcaggtgtgaagt-3′.

### Real-time reverse transcription-PCR (real-time RT-PCR)

Total RNA was isolated from DPSCs using TRIzol reagent (Invitrogen, Carlsbad, CA, USA). A 2 μg RNA aliquot was used as a template to synthesize cDNA by using oligo (dT) and reverse transcriptase, according to the manufacturer's protocol [23]. Next, real-time RT-PCR was performed according to the QuantiTect SYBR Green PCR kit (Qiagen, Hilden, Germany) and an IcycleriQ Multi-colour Real-time RT-PCR Detection System instructions [23]. The primers for specific genes used in this study are protected by copyright. All rights reserved. The list of primer sequences is presented in Additional file 2: Table 1. The relative gene expression data was analyse by the 2^−△△CT^ method.

### Alkaline phosphatase (ALP) activity assay and Alizarin red detection

DPSCs were seed to culture in routine medium, incubated at 37℃ in 5% carbon dioxide. When the DPSCs grown to 80% confluence, we replaced the routine medium with osteo/dentinogenic induced medium by using the osteogenic‐inducing medium g CA), which contains 100 μM/mL ascorbic acid, 2 mM β‐glycerophosphate, 1.8 mM KH2PO4 and 10 nM dexamethasone and ALP activity was measured using an ALP activity kit (Sigma-Aldrich, St. Louis, MO, USA) according to the manufacturer's protocol [28]. After osteogenic induction of DPSCs for 2 weeks, 70% ethanol and 2% Alizarin red (Sigma-Aldrich, St. Louis, MO, USA) were used to fix and stain the cultured cells. The plates were then destained for 30 min at room temperature with 10% cetylpyridinium chloride [28]. The absorbance of the cell cultures was measured at 562 nm on a multi‐plate reader, and the final calcium level was normalized to the total protein concentration in duplicate plates.

### Western blot analysis

Total protein extraction and SDS-polyacrylamide gel electrophoresis procedures were performed as described in a previous study [22, 23]. Immune complexes on membranes were incubated with horseradish peroxidase‐conjugated anti‐ rabbit or anti‐mouse IgG (Promega, Madison, WI) and visualized with SuperSignal reagents (Pierce, Rockford, IL). The primary antibodies used in this study were anti‐EREG (Cat No. 93815; Cell Signaling Technology, Boston, MA, USA), mouse monoclonal anti‐HA (Clone No. C29F4; Cat No. MMS‐101P; Covance, Princeton, NJ, USA), anti‐phospho‐p38 MAPK (Cat No. 4631; Cell Signaling Technology, Boston, MA, USA), anti‐p38 MAPK (Cat No. 8690; Cell Signaling Technology, Boston, MA, USA), anti‐phospho‐Erk1/2 (Cat No. 4377S; Cell Signaling Technology, Boston, MA, USA), and anti‐Erk1/2 (Cat No. 4695S; Cell Signaling Technology, Boston, MA, USA). The primary monoclonal antibody used as a housekeeping control was a monoclonal antibody against β‐actin (Cat No. C1313; Applygen, Beijing, China).

### Statistical analysis

Each experiment was independently conducted at least three times. The results are expressed as the mean ± standard deviation (SD). Significant differences were assessed by Student’s t test (two-tailed) and one-way ANOVA with the post hoc Bonferroni test. *P* ≤ 0.05 was considered significant.

## Results

### Knockdown of EREG enhanced the osteo/dentinogenic differentiation of DPSCs

**Fig. 1:**
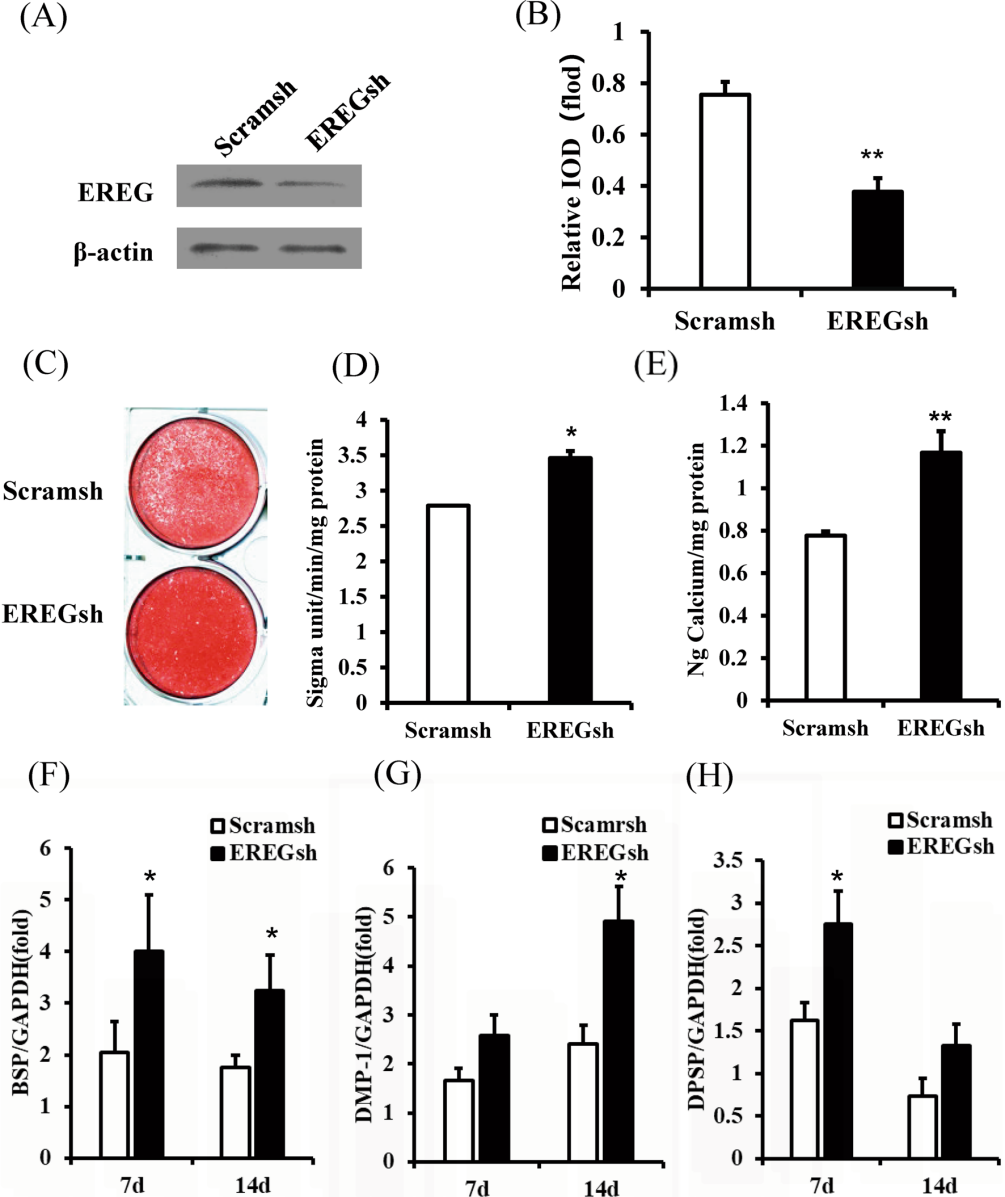
Knockdown of EREG enhanced the osteo/dentinogenic differentiation ability of DPSCs. Short hairpin RNAs were used to silence the expression of EREG (EREGsh), and scramble shRNA was used as a control (Scramsh). **a** Western blot results for the expression of EREG in EREGsh and Scramsh DPSCs. **b** Real time PCR results for the expression of EREG in EREGsh and Scramsh DPSCs. **c**Alizarin red staining results for both groups after 2 weeks of osteogenic induction. **d** ALP activity results of EREGsh and Scramsh DPSCs on day 5. **e** Calcium quantitative analysis results for both groups after 2 weeks of osteogenic induction. **f**–**g** Real-time RT-PCR for the expression of the osteo/dentinogenic differentiation markers BSP, DMP-1 and DSPP. GAPDH was used as the internal control. Error bars represent the SD (n = 3). Student's t test was used to test statistical significance. *P ≤ 0.05. **P ≤ 0.01

To investigate the function of EREG on DPSCs, we knocked down EREG expression in DPSCs using lentiviral vector infection (EREGsh), and Scramsh was used as control. The knocking down efficiency was over 60% tested by Western blot and real time PCR (Fig. 1a, b). Further, we studied the effect of EREG on the osteo/dentinogenic differentiation potentials of DPSCs. Transduced DPSCs were cultured in osteo/dentinogenic-inducing medium, and the results indicated that EREG depletion enhanced the ALP activity of DPSCs compared with the Scramsh group (Fig. 1d). Two weeks after induction, Alizarin red staining and quantitative calcium measurements revealed increased mineralization in vitro in EREGsh DPSCs compared with Scramsh DPSCs (Fig. 1c, e). Real-time RT-PCR was conducted on days 7 and 14 during osteo/dentinogenic induction, and the results revealed that the level of the osteo/dentinogenic marker gene BSP significantly increased on days 7 and 14 (Fig. 1f) in EREGsh DPSCs compared with Scramsh DPSCs. In addition, the DMP-1 expression level was clearly elevated on day 14 (Fig. 1g), and the DSPP expression level was elevated on day 7 (Fig. 1h).

### EREG recombinant protein inhibited the osteo/dentinogenic differentiation of DPSCs

**Fig. 2:**
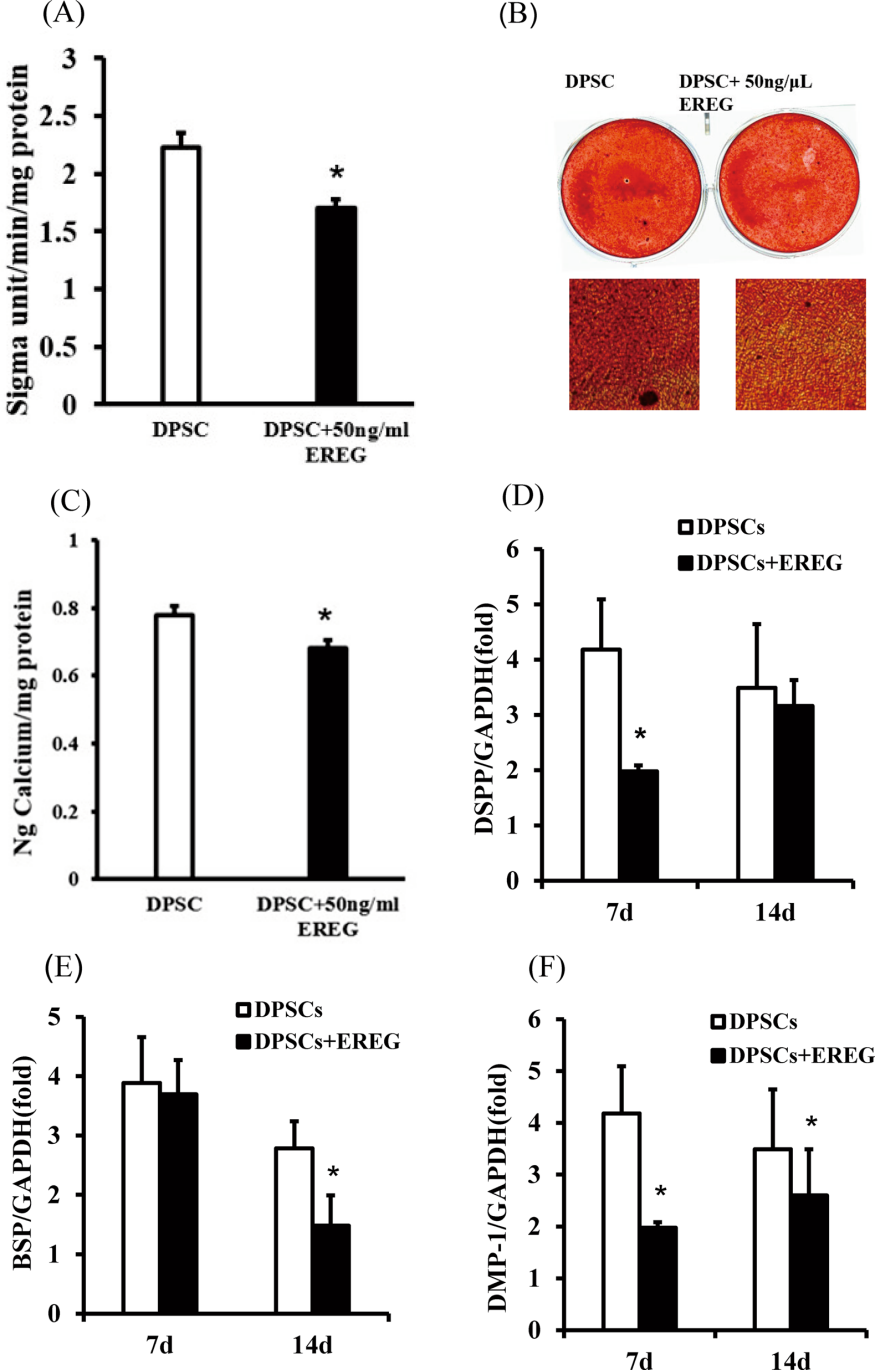
rhEREG inhibited the osteo/dentinogenic differentiation ability of DPSCs. **a** ALP activity results of the control and EREG-treated groups on day 5. **b**, **c** Alizarin red staining and calcium quantitative analysis results for both groups after 2 weeks of osteogenic induction. **d**–**f** Real-time RT-PCR for the expression of the osteo/dentinogenic differentiation markers DSPP, BSP and DMP-1. GAPDH was used as the internal control. Error bars represent the SD (n = 3). Student's t test was used to test statistical significance. *P ≤ 0.05. **P ≤ 0.01

To further verify the effect of EREG on the DPSC osteo/dentinogenic potential, Exogenous rhEREG protein of different concentration (25, 50, 100 ng/ml) was added into the culture medium and ALP activity was measured to evaluate the effect of different concentration of rhEREG on DPSCs. The result showed that the concentration of 50 ng/ml displayed evident effect on ALP activity than other concentrations. Thus, 50 ng/ml was chosen for the following study. (Additional file 1: Figure 1). Then, 50 ng/mL rhEREG protein was added during osteo/dentinogenic induction, and mineralization ability was determined. After 5 days of osteogenic induction, the ALP activity assay results showed that 50 ng/mL EREG decreased ALP activity (Fig. 2a). Two weeks later, Alizarin red staining and the quantitative calcium analysis results indicated that 50 ng/mL EREG impaired the mineralization ability of DPSCs (Fig. 2b, c). In addition, real-time RT-PCR results showed that DSPP expression was significantly decreased on day 7 (Fig. 2d), BSP expression decreased on day 14 (Fig. 2e) and DMP-1 expression decreased significantly on both day 7 and 14 (Fig. 2f) when comparing EREG-treated DPSCs with control DPSCs.

### EREG depletion activated the p38 MAPK and Erk signalling pathways in DPSCs

**Fig. 3:**
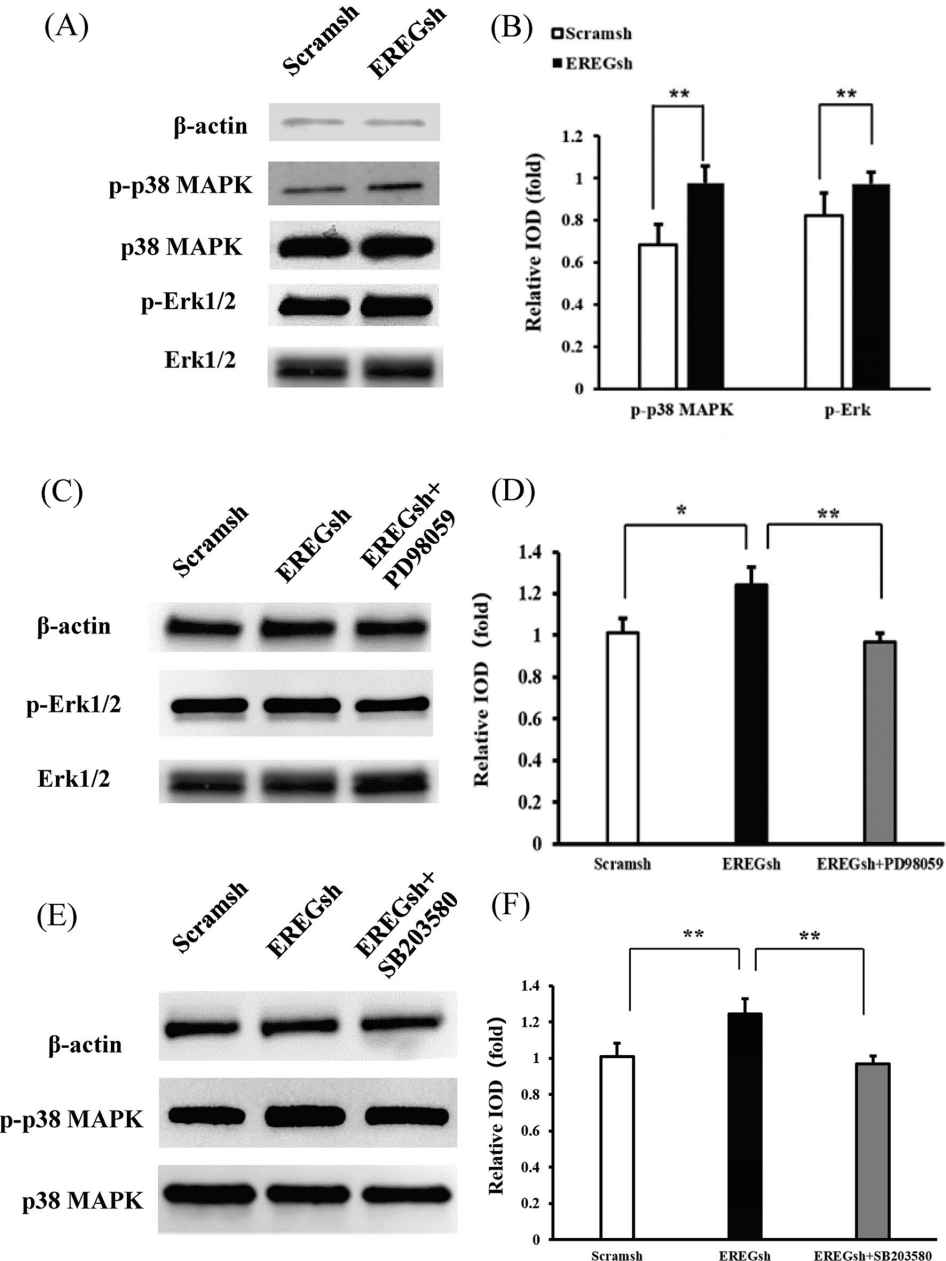
Knockdown of EREG activated the p38 MAPK and Erk signalling pathways in DPSCs. **a**, **b** Western blot results and quantitative analysis of the expression of phosphorylated p38 MAPK and phosphorylated Erk in EREGsh and Scramsh DPSCs. β-Actin was used as an internal control. **c**, **d** Western blot results and quantitative analysis of the expression of phosphorylated Erk in DPSCs after treatment with 20 μmol/L Erk‐specific inhibitor PD98059 for 1 h in DPSCs. β-Actin was used as an internal control. **e**, **f** Western blot results and quantitative analysis of the expression of phosphorylated p38 MAPK in DPSCs after treatment with 20 μmol/L p38 MAPK‐specific inhibitor SB203580 for 1 h in DPSCs. β-Actin was used as an internal control. Error bars represent the SD (n = 3). One-way ANOVA with the post hoc Bonferroni test were used to test statistical significance. *P ≤ 0.05. **P ≤ 0.01

In the following study, we explored the mechanism by which EREG affects the osteo/dentinogenic potential of DPSCs. Western blotting was carried out to evaluate the protein levels of critical members of the MAPK pathway. The results showed that silencing EREG upregulated the expression of phosphorylated p38 MAPK and Erk1/2 in DPSCs, since the levels of phosphorylated p38 MAPK and Erk1/2 were increased, while the levels of total p38 MAPK and Erk1/2 were not affected (Fig. 3a, b). To further validate our findings, Erk1/2 and p38 MAPK were inhibited with the specific inhibitors PD98059 and SB203580, respectively. DPSCs with EREG depletion were pretreated with 20 μmol/L of either inhibitor for 1 h. The Erk1/2-specific inhibitor PD98059 effectively suppressed the expression of phosphorylated Erk1/2 in EREGsh DPSCs (Fig. 3c, d). The p38 MAPK‐specific inhibitor SB203580 displayed similar results (Fig. 3e, f). These findings indicated that the effect of EREG on the osteo/dentinogenic differentiation of DPSCs was probably dependent on the regulation of the p38 MAPK and Erk signalling pathways.

### EREG depletion enhanced the osteo/dentinogenic differentiation potential of DPSCs after stimulation with TNF‐α

**Fig. 4:**
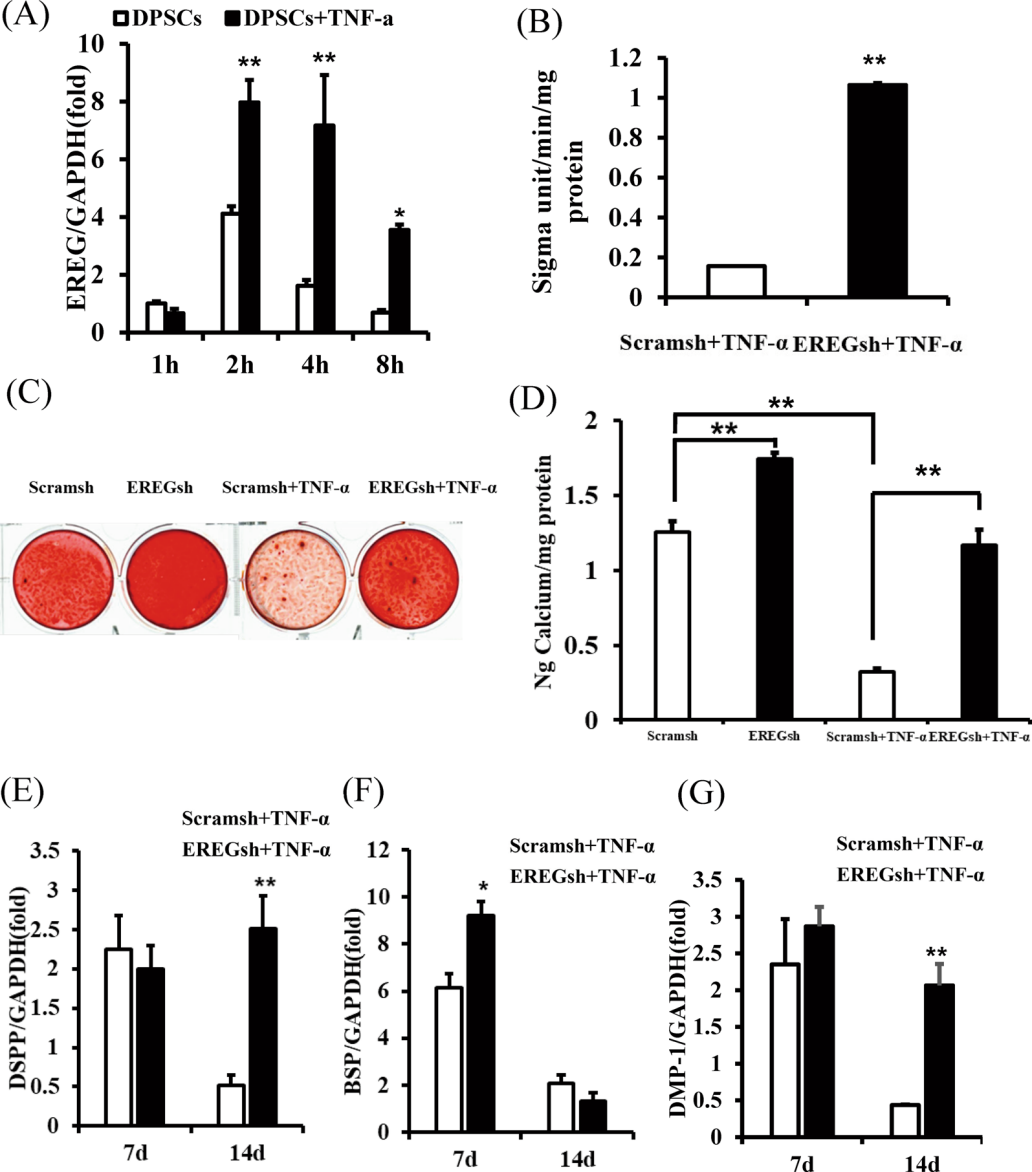
Knockdown of EREG improved the osteo/dentinogenic differentiation ability of DPSCs under TNF-α stimulation. DPSCs were treated with 10 ng/mL TNF‐α. **a** Real-time RT-PCR results for the expression of EREG at 1, 2, 4 and 8 h after 10 ng/mL TNF‐α treatment of DPSCs. **b** ALP activity results of EREGsh and Scramsh DPSCs under TNF-α stimulation. **c**, **d** Alizarin red staining and calcium quantitative analysis results for EREGsh and Scramsh DPSCs under TNF-α stimulation. (E–G): Real-time RT-PCR results for the expression of DSPP, BSP and DMP-1 in EREGsh and Scramsh DPSCs under TNF-α stimulation. GAPDH was used as the internal control. Error bars represent the SD (n = 3). Student's t test were used to test statistical significance. *P ≤ 0.05. **P ≤ 0.01

We then studied the effect of EREG on DPSCs in inflammatory microenvironment. TNF‐α (10 ng/mL) was used to mimic the inflammatory microenvironment in vitro, and EREG levels were quantified. The real‐time RT‐PCR results showed that EREG expression increased at 2, 4 and 8 h after 10 ng/mL TNF‐α treatment (Fig. 4a). Furthermore, we investigated the function of EREG in DPSCs upon 10 ng/mL TNF‐α treatment. EREGsh cells in TNF-α treatment promoted ALP activity and mineralization ability than those of scramsh cells (Fig. 4b–d). Similarly, real‐time RT‐PCR showed that with the treatment of TNF-α, EREG-knockdown enhanced the expression levels of DSPP, DMP-1 and BSP after 2 weeks osteogenic induction (Fig. 4e–g).

### EREGsh activated the p38 MAPK and Erk1/2 signalling pathways that were inhibited by TNF‐α stimulation

**Fig. 5:**
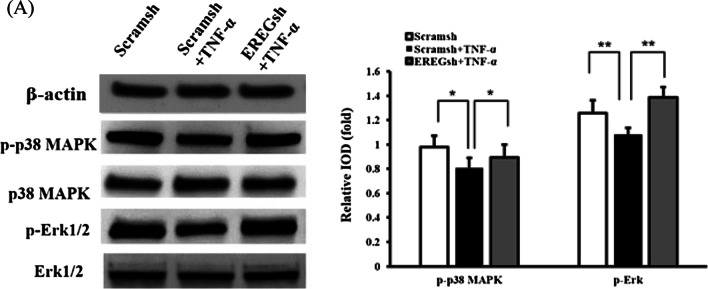
EREG depletion restored the decreased phosphorylation of p38 MAPK and Erk1/2 in TNF‐α-stimulated DPSCs. DPSCs were treated with 10 ng/mL TNF‐α for 4 h. **a** Western blot results and quantitative protein analysis of the expression of phosphorylated p38 MAPK and Erk1/2. β-Actin was used as an internal control. Error bars represent the SD (n = 3). One-way ANOVA with the post hoc Bonferroni test were used to test statistical significance. *P ≤ 0.05. **P ≤ 0.01

We further investigated the effect of EREG on the p38 MAPK and Erk signalling pathways under inflammatory conditions. Western blot analysis revealed that the levels of phosphorylated p38 MAPK and phosphorylated Erk1/2 were decreased in DPSCs after 10 ng/mL TNF‐α stimulation (Fig. 5a), and knockdown of EREG reversed these effects.

## Discussion

The use of MSCs for pulp-dentin regeneration brings hope for the treatment of pulpitis and functional reconstruction of the pulp-dentin complex. However, the local inflammatory microenvironment is one of the key factors affecting the efficiency of stem cell-mediated tissue regeneration.

In the present study, we found that EREG depletion promoted the osteo/dentinogenic differentiation potential of DPSCs. Accordingly, rhEREG exerted the opposite effect. This result is contrary to our previous findings, which indicated that EREG could improve the osteogenic differentiation of SCAPs and ADSCs [22, 23]. Similarly, a recent study illustrated that EREG could enhance the odontoblastic differentiation of DPSCs [29]. The reasons why EREG has different effects in different studies may be based on the following aspects. First, different cell types may respond differently to EREG. Furthermore, even for a specific cell type, the effect of EREG depends on its concentration. High concentrations (100 ng/mL) increased the osteogenic differentiation activity of DPSCs in Cui’s study, while a lower concentration (50 ng/mL) of EREG inhibited the osteogenic differentiation of DPSCs in the present study. Thus, we speculated that EREG is a concentration-sensitive cytokine. Finally, the use of different experimental tools may also lead to different results. In the present study, the primer sequence for EREG shRNA was 5′-actactgcaggtgtgaagt-3′, while in Cui’s study, the primer sequence was 5′-ggctttgaccgtgattcttat-3′. The use of different sequences may result in different knockdown efficiencies of EREG, or targeting different functional regions may generate different outcomes. In fact, as a growth factor, EREG could bind with epidermal growth factor receptor (EGFR) to regulate various biological processes, including cell growth, motility, proliferation and differentiation [30, 31] EREG can be released from vascular smooth muscle cells and acts as a major autocrine/paracrine factor for dedifferentiation [32]. EREG could also stimulate the proliferation of fibroblasts, hepatocytes, and smooth muscle cells [33, 34]; conversely, EREG may inhibit the expansion of several types of tumour-derived epithelial cells [35, 36]. Thus, EREG may exert diverse effects depending on the dose and the cell type.

In this study, we further investigated the effects of EREG on DPSCs under inflammatory conditions. Studies have shown that inflammatory factors such as TNF-α, IL-6 [37] and IL-8 [38] are closely related to the development of pulpitis [39]. In clinical research, the amount of TNF-α in dental pulp tissue of pulpitis was much higher than that in normal pulp tissue [40, 41]. Studies have revealed that TNF-α inhibits osteoblastogenesis through several mechanisms [42]. However, it has also been reported that TNF-α promotes osteoblastogenesis [43]. These discrepancies may depend on the cellular type, concentration, timing and duration of TNF-α administration. In our study, TNF‐α was used to mimic inflammatory conditions and under stimulation with 10 ng/mL TNF-α [44], EREG expression was found to increase. EREG depletion rescued the osteo/dentinogenic differentiation of DPSCs that was impaired by TNF-α. The present study indicated that EREG may act as a new target to regulate the osteo/dentinogenic differentiation of DPSCs under inflammatory conditions.

A previous study has found that TNF‐α stimulation suppressed the p38 MAPK, JNK and Erk1/2 signalling pathways in periodontal ligament stem cells (PDLSCs) [28]. Nevertheless, other studies demonstrated that TNF-α mediates p38 MAPK activation and negatively regulates bone formation [45]. Our investigation showed that TNF-α inhibited the p38 MAPK and Erk1/2 signalling pathways; however, EREG depletion reversed this effect. EREG is one of the most potent ligands that binds EGF receptors [14]. and thus couples to numerous signalling cascades, including MAPKs. MAPK signalling, which consists of p38 MAPK, JNKs, and Erk1/2, involves a set of serine/threonine kinases and plays a crucial role in various physiological functions [46] p38 MAPK and Erk1/2 can be stimulated by inflammation, hypoxia and environmental stress changes. Studies have highlighted the involvement of EREG and the MAPK-Erk1/2 signalling pathway activated by *Streptococcus suis* serotype 2 in the subsequent initiation and regulation of the inflammatory response in the brain and finally CNS dysfunction [47]. In addition, rhinovirus RV16 infection could rapidly promote the induction of EREG, thus increasing IL-8 and ICAM-1 levels through the p38 MAPK and Erk1/2 pathways [25]. Under stimulation with the proinflammatory cytokine PGE2, human granulosa cells may induce the biosynthesis of EREG, which further activates the MAPK pathways [48]. Besides, the activation of Erk1/2 signalling is required during cytokine-mediated osteogenic differentiation in PDLSCs [49], SCAPs [22], and bone marrow stem cells (BMSCs) [50], as well as stem cell homing/migration [23]. Consistent with our previous study, the present study demonstrated the involvement of both Erk1/2 and p38 MAPK signalling in the osteo/dentinogenic differentiation of DPSCs.

Studies have shown that the Erk signalling pathway is a downstream molecule of the p38 MAPK signalling pathway. Other studies about the effects of Erk1/2 and p38 on the osteo/dentinogenic differentiation of DPSCs have indicated that inhibition of p38 MAPK suppresses the osteogenic differentiation of DPSCs, whereas inhibition of Erk1/2 demonstrates the opposite effect [43]. Different chemical and physical stimuli, cell types, culture methods, times of inhibitor administration and dosages of the inhibitor may influence the effect of Erk1/2 and p38 on the differentiation of MSCs. In the present study, we used specific inhibitors to block the p38 MAPK and Erk 1/2 signalling pathway and found that depletion of EREG could enhance the expression of both pathways. However, the correlation and effect of the two pathways on the osteo/dentinogenic differentiation of DPSCs needs further study.

## Conclusion

Our study demonstrated that EREG negatively regulated the osteo/dentinogenic differentiation potential of DPSCs via the p38 MAPK and Erk pathways. Under inflammatory environment, EREG depletion could rescue the osteo/dentinogenic differentiation potential of DPSCs by improving the expression of p-p38 MAPK and p-Erk. These results provide insights into the mechanisms underlying the functional regulation of DPSCs and indicate that EREG has a significant role in maintaining the osteo/dentinogenic differentiation potential of DPSCs.

## Supplementary Information

**Additional file 1**. 50ng/ml recombinant protein inhibited the osteo/dentinogenic differentiation of DPSCs.

**Additional file 2**. The primers for specific genes used in Real-time PCR.

## Data Availability

The sequences generated during the current study are available in the NCBI repository[https://www.ncbi.nlm.nih.gov/gene/2069]. The other datasets used and/or analysed during the current study available from the corresponding author on reasonable request.

## Abbreviations

ALP: Alkaline phosphatase
α-MEM: α Minimal essential medium
BMSCs: Bone marrow stromal cells
BSP: Bone sialoprotein
CNS: Central nervous system
DPSCs: Dental pulp stem cells
DMSO: Dimethyl sulfoxide
DSPP: Dentin sialophosphoprotein
DMP-1: Dentin matrix protein-1
EGF: Epidermal growth factor
EREG: Epiregulin
Erk: Extracellular signal-regulated protein kinases
FBS: Fetal bovine serum
GAPDH: Glyceraldehyde-3-phosphate dehydrogenase
HB-EGF: Heparin-binding epidermal growth factor
ICAM-1: Intercellular cell adhesion molecule-1
IL-6: Interleukin-6
IL-8: Interleukin-8
JNK: C-Jun N-terminal kinase
MAPK: Mitogen- activated protein kinase
MSCs: Mesenchymal stem cells
OD: Optical density
PCR: Polymerase chain reaction
PDLSCs: Periodontal ligament stem cells
PGE2: Prostaglandin E2
rhEREG: Recombinant human EREG protein
RT-PCR: Reverse transcriptase-polymerase chain reaction
SCAPs: Apical papilla
shRNA: Short hairpin RNAs
TGF-α: Transforming growth factor alpha
TNF-α: Tumor necrosis factor alpha
