# Supplementary material for: Depletion of EREG enhances the osteo/dentinogenic differentiation ability of dental pulp stem cells via the p38 MAPK and Erk pathways in an inflammatory microenvironment
Source: BMC Oral Health. 2021 Jun 21;21:314. doi: 10.1186/s12903-021-01675-0 (PMC8215766; doi:10.1186/s12903-021-01675-0)
Supplement: Supplementary file 1 — Additional file 1. 50ng/ml recombinant protein inhibited the osteo/dentinogenic differentiation of DPSCs. [file 12903_2021_1675_MOESM1_ESM.pdf]

Depletion of EREG enhances the osteo/dentinogenic differentiation ability of dental pulp stem cells via p38 MAPK and Erk pathway in inflammatory microenvironment

Running head: EREG inhibits osteo/dentinogenic differentiation of DPSC

Ran Ran12 | Haoqing Yang1 | Yangyang Cao1| Wanhao Yan1 | Luyuan Jin3* | Ying Zheng2*

1 Laboratory of Molecular Signaling and Stem Cells Therapy, Beijing Key Laboratory of Tooth Regeneration and Function Reconstruction, Capital Medical University, School of Stomatology, Beijing, China.

2 Department of Endodontics, Capital Medical University School of Stomatology, Beijing, China.

3 Department of General Dentistry and Integrated Emergency Dental Care, Capital Medical University, School of Stomatology, Beijing, China.

* Correspondence

Dr.Ying Zheng, Department of Endodontics, Capital Medical University School of Stomatology, Beijing 10050,China.Tel.:+86 10 5709 9074; Fax:861067062012; Email:zhengyingyus@163.com

Or Dr. Luyuan Jin, Department of General Dentistry and Integrated Emergency Dental Care, Capital Medical University School of Stomatology, Beijing 100050, China. Tel.: +86 10 5709 9255; Fax: 861067062012; E-mail: sujin_0309@163.com.

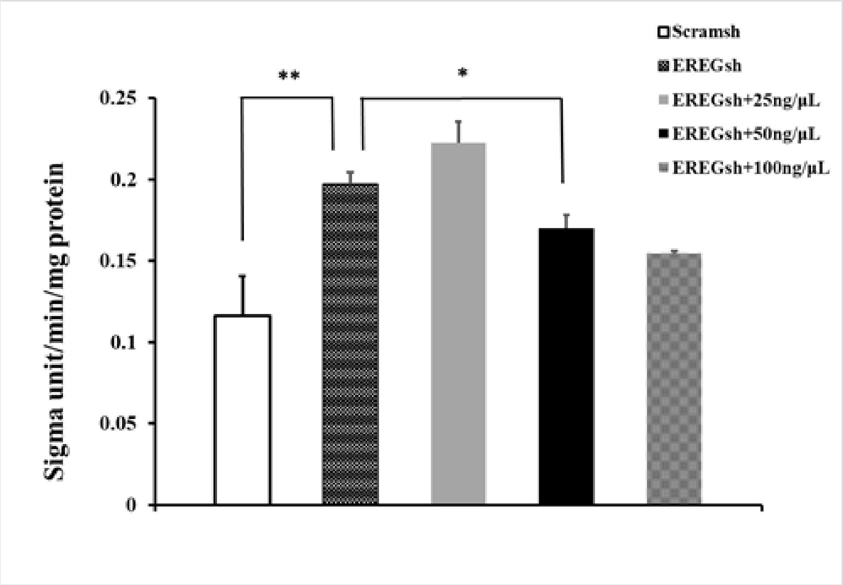
Scramsh
EREGsh
EREGsh+25ng/µL
EREGsh+50ng/µL
EREGsh+100ng/µL
**
*
Sigma unit/min/mg protein
0.25
0.2
0.15
0.1
0.05
0

Figure 1

Figure 2

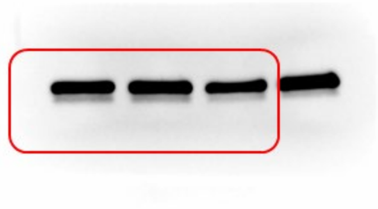

Figure3(B)-p-Erk

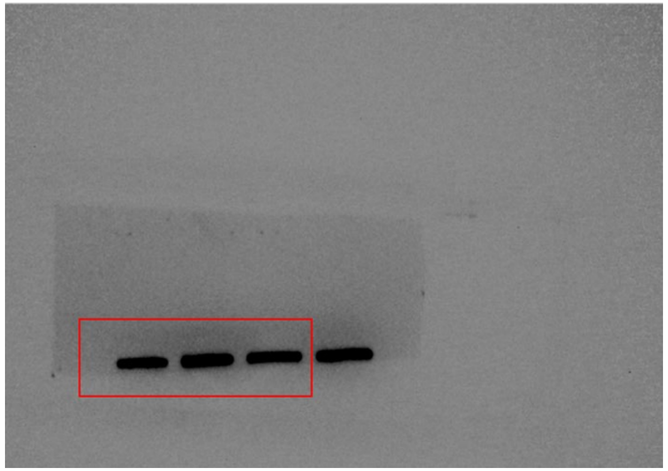

Figure3(B)-p-p38

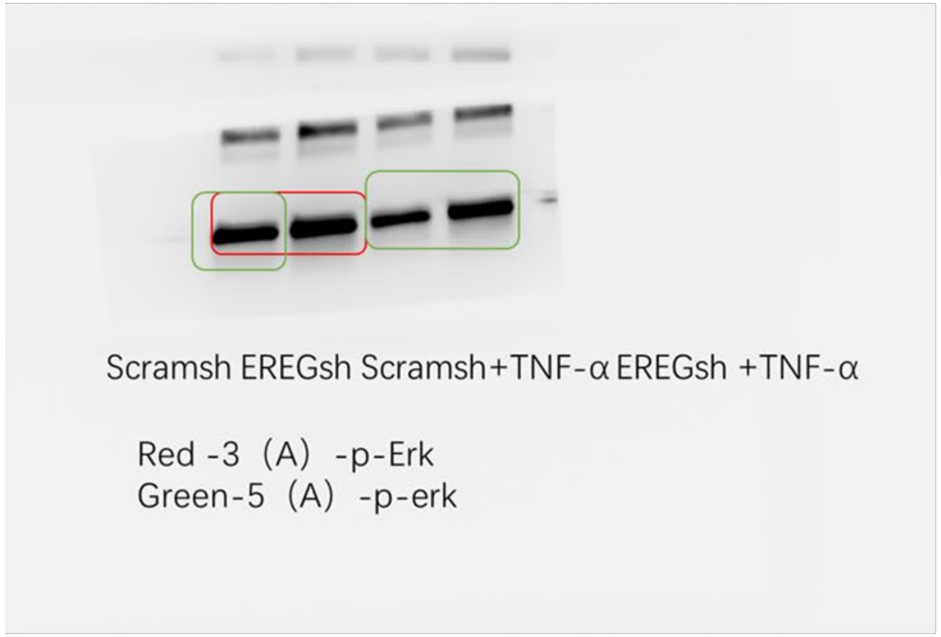

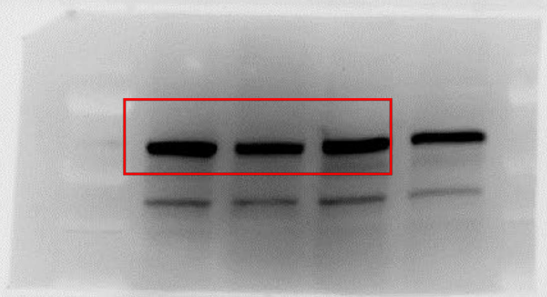

Figure-5 (A) p-p38
