# Supplementary material for: Depletion of EREG enhances the osteo/dentinogenic differentiation ability of dental pulp stem cells via the p38 MAPK and Erk pathways in an inflammatory microenvironment
Source: BMC Oral Health. 2021 Jun 21;21:314. doi: 10.1186/s12903-021-01675-0 (PMC8215766; doi:10.1186/s12903-021-01675-0)
Supplement: Supplementary file 2 — Additional file 2. The primers for specific genes used in Real-time PCR. [file 12903_2021_1675_MOESM2_ESM.doc]

**Depletion of EREG enhances the osteo/dentinogenic differentiation ability of dental pulp stem cells via the p38 MAPK and Erk pathways in an inflammatory microenvironment**

Running head: EREG inhibits the osteo/dentinogenic differentiation of DPSCs

Ran Ran^12^ | Haoqing Yang^1^ | Yangyang Cao^1^| Wanhao Yan^1^ | Luyuan Jin^3^* | Ying Zheng^2^*

1 Laboratory of Molecular Signaling and Stem Cells Therapy, Beijing Key Laboratory of Tooth Regeneration and Function Reconstruction, Capital Medical University, School of Stomatology, Beijing, China.

2 Department of Endodontics, Capital Medical University School of Stomatology, Beijing, China.

3 Department of General Dentistry and Integrated Emergency Dental Care, Capital Medical University, School of Stomatology, Beijing, China.

* Correspondence

Dr.Ying Zheng, Department of Endodontics, Capital Medical University School of Stomatology, Beijing 10050,China.Tel.:+8610 5709 9074; Fax:861067062012; Email:zhengyingyus@yahoo.com

Or Dr. Luyuan Jin, Department of General Dentistry and Integrated Emergency Dental Care, Capital Medical University School of Stomatology, Beijing 100050, China. Tel.: +86 10 5709 9255; Fax: 861067062012; E-mail: [sujin_0309@163.com](mailto:sujin_0309@163.com).

**Supplementary table 1. The primers for specific genes used in Real-time RT-PCR**

| **Specific Genes** | **Target Sequences** |
| --- | --- |
| GAPDH-Forward  GAPDH-Reverse  BSP-Forward  BSP-Reverse  DSPP-Forward  DSPP-Reverse  DMP1-Forward  DMP1-Reverse  EREG-Forward  EREG-Reverse | 5’‑CGGACCAATACGACCAAATCCG-3’ 5’‑AGCCACATCGCTCAGACACC-3’ 5’‑CAGGCCACGATATTATCTTTACA-3’ 5’‑CTCCTCTTCTTCCTCCTCCTC-3’ 5’‑CGACATAGGTCACAATGAGGATGTCG-3’ 5’‑TTGCTTCCAGCTACTTGAGGTC-3’ 5’‑CGTGGACAAAGAAGATAGCAACTCCACG-3’ 5’‑TTCCGGCTCTCTATCTCAATGTTT-3’  5’‑TTATGGGAGGCTCCTTCATC-3’  5’‑GCCTTCGTTTACCCTAGCAC-3’ |
